# Supplementary figures and images for: bsAS, an antisense long non-coding RNA, essential for correct wing development through regulation of blistered/DSRF isoform usage
Source: PLoS Genet. 2020 Dec 28;16(12):e1009245. doi: 10.1371/journal.pgen.1009245 (PMC7793246; doi:10.1371/journal.pgen.1009245)

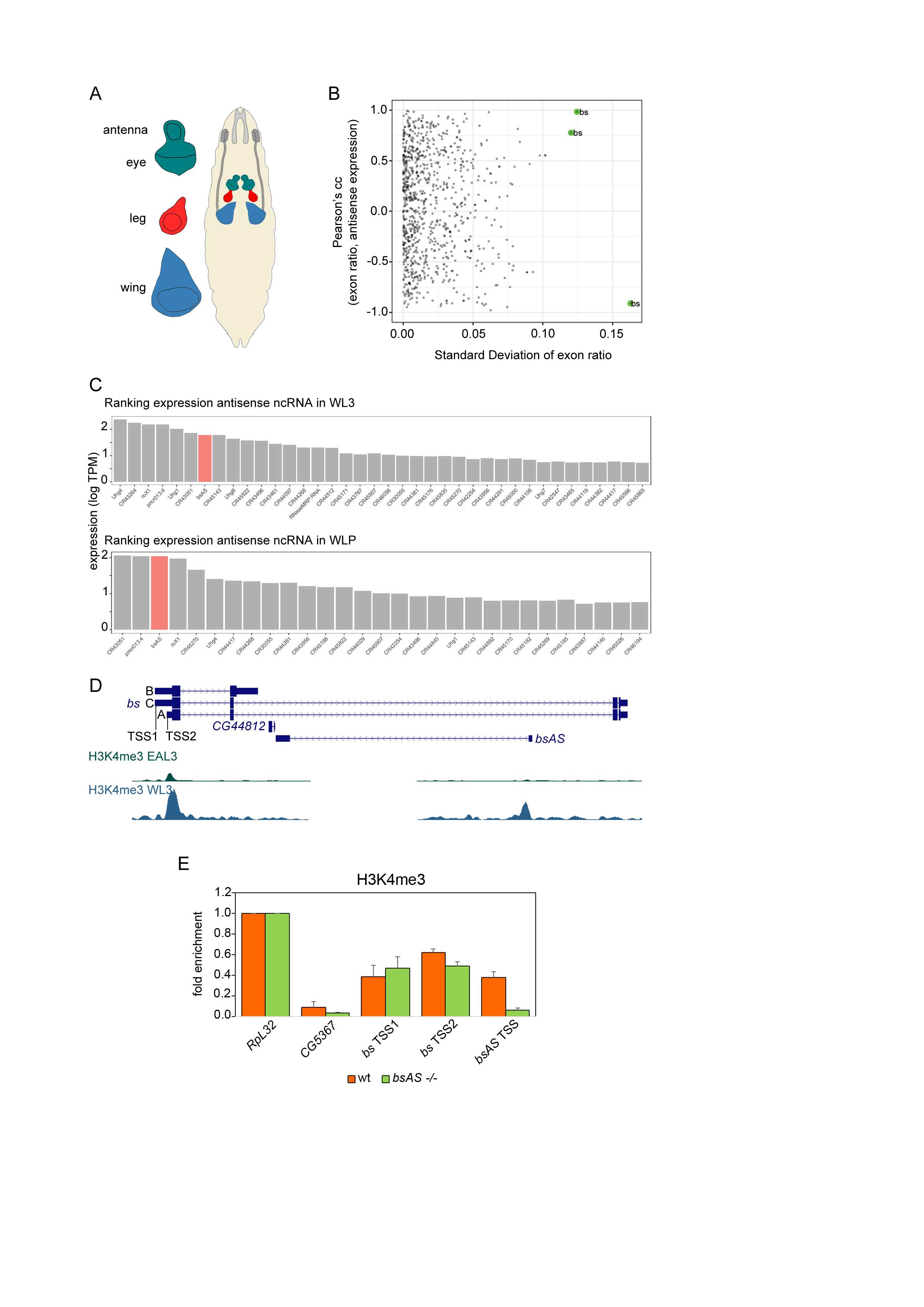

Supplement: S1 Fig — (A) Third instar larvae tissues used for RNA-Seq experiments. (B) Relationship between the correlation of exon ratio and antisense expression and the standard deviation (st. dev.) of sense gene exons ratio. Of the 145 SA pairs with both genes expressed in at least one tissue in L3, 104 pairs (72%, 102 genes) involve a protein coding gene with multiple isoforms. We computed the correlation between the expression of these NATs and the inclusion of the 964 exons of the sense protein coding genes. Because of the very small number of independent data points, we have little power to detect significant correlations. Still, we plotted this correlation against the standard deviation of the exon inclusion values, to focus specifically in the exons that changed the most (see Material and Methods). Among them, the case of blistered -bs- is the strongest, as it indeed shows strong positive and negative correlation between the expression of the NAT antisense to bs and the inclusion of three highly variable bs exons. (C) Ranking of expression of antisense lncRNAs in L3 (upper panel) and LP (lower panel) wings. bsAS is highlighted in red. (D) Histone methylation marks [77] at bs locus. There is a strong peak of H3K4me3 at bs TSS and bsAS TSS in WL3, whereas only a small peak at bs TSS is observed in EAL3. (E) H3K4me3 ChIP-qPCR in WL3. No differences are observed between bs TSS1 and TSS2 H3K4me3 marking. The deletion of bsAS TSS induces a dramatic reduction of H3K4me3 marking in bsAS TSS. TSS1 and TSS2 of bs do not show differences between bsAS-/- and wt. RpL32 and CG5367 correspond to positive and negative controls of the ChIP, respectively. (TIF) [file pgen.1009245.s001.tif]

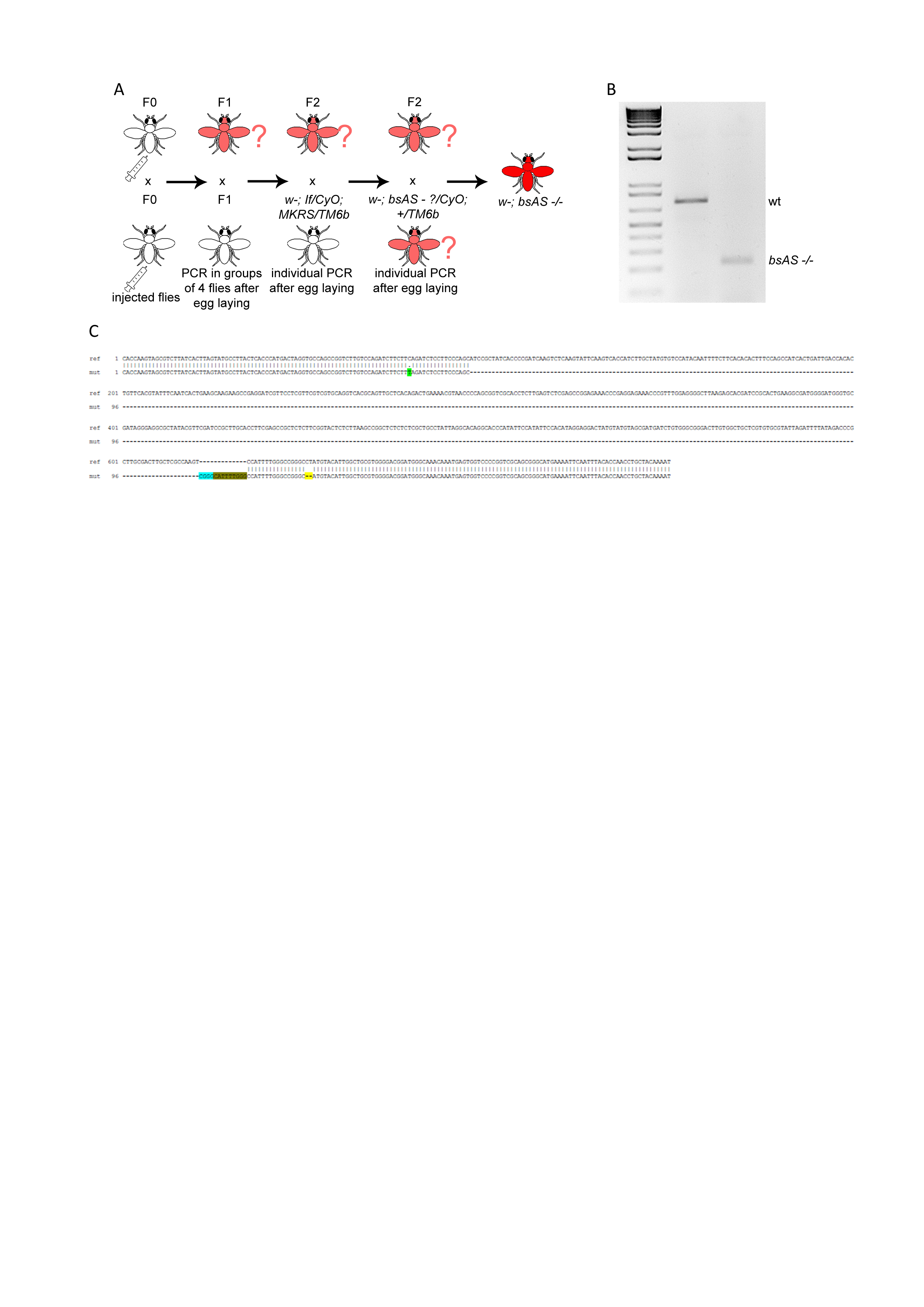

Supplement: S2 Fig — (A) Drosophila embryos expressing Cas9 nuclease in the germinal cells under the control of vasa driver, were injected with a mix of two plasmids expressing gRNAs against the TSS of bsAS. The screening was performed retroactively, by allowing putatively mutant flies to lie eggs before screening. Once the mutation was isolated, genetic crosses were performed to obtain the homozygous mutant flies. (B) PCR screening of bsAS deletion. A band of 800 bp was amplified in wt flies, whereas a band of 250 bp was obtained from homozygous bsAS mutant flies. (C) Alignment of wt and bsAS-/- genomic regions. A Single Nucleotide Polymorphism was detected in the bsAS TSS sequence (in green). The 5’ region of the deletion has been cleanly repaired, but in the 3’ region, several insertions/deletions have occurred (the insertion of 4 nucleotides–in blue-, the duplication of 9 nucleotides–in green- and the deletion of 2 nucleotides–in yellow-). (TIF) [file pgen.1009245.s002.tif]

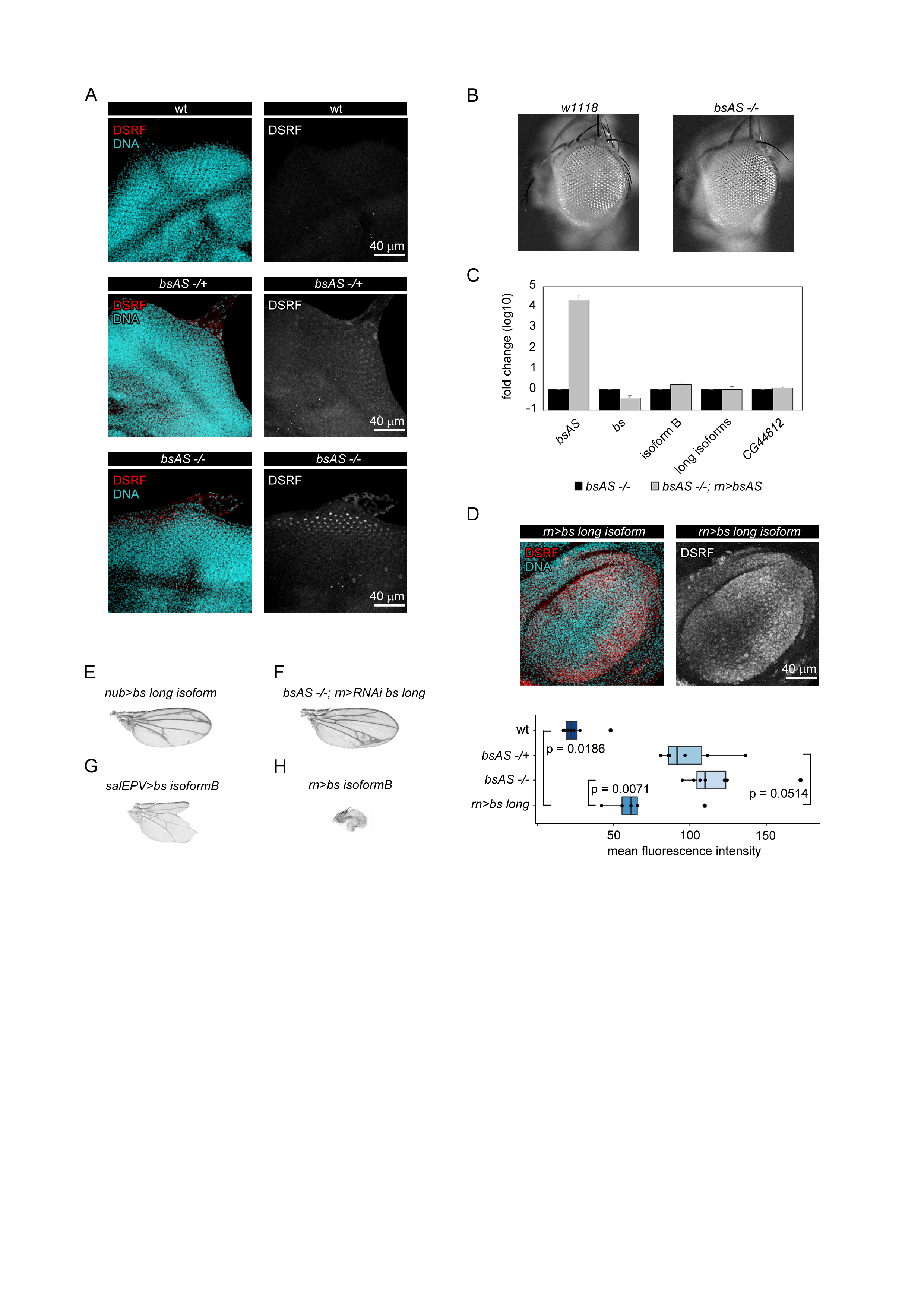

Supplement: S3 Fig — (A) Expression pattern of DSRF in bsAS mutant eye imaginal discs. Immunostaining of DSRF (red and grey) in wt (upper panels), bsAS -/+ (middle panels) and bsAS -/- (lower panels) EL3. DSRF is overexpressed in a subset of cells posterior to the morphogenetic furrow in homozygous bsAS mutants. (B) Eyes of w1118 (left panel) and bsAS -/- (right panel) adult males. No evident phenotype is observed in adult eyes, despite the overexpression of DSRF in third instar larvae. (C) Overexpression of bsAS under the control of rotund (rn) driver (specific driver that induces GAL4 expression in the wing pouch) in a bsAS mutant background, checked by qPCR. The overexpression of bsAS does not change the expression of any of bs isoforms. (D) DSRF staining on third instar larvae wings overexpressing the long isoform of bs under the control of rn driver. The overexpression of DSRF protein in L3 wings is significantly lower in wings overexpressing the long isoform of bs than in bsAS mutants. (E-H) Adult wings from males. (E) Wings overexpressing bs long isoform A under the control of the nub driver. They present extra vein tissue. (F) Wings overexpressing RNAi specific against the long isoform of bs in a bsAS homozygous mutant background. They show a partial rescue of the mutant phenotype, being less creased (compare to Fig 2G). (G) Wings expressing the short isoform of bs under the control the salE/Pv driver. They present notches in the wing margin. (H) Wings expressing the short isoform of bs under the control the rn driver. The overexpression of the isoform B of bs induces high lethality and strong impairment of wing development. (TIF) [file pgen.1009245.s003.tif]

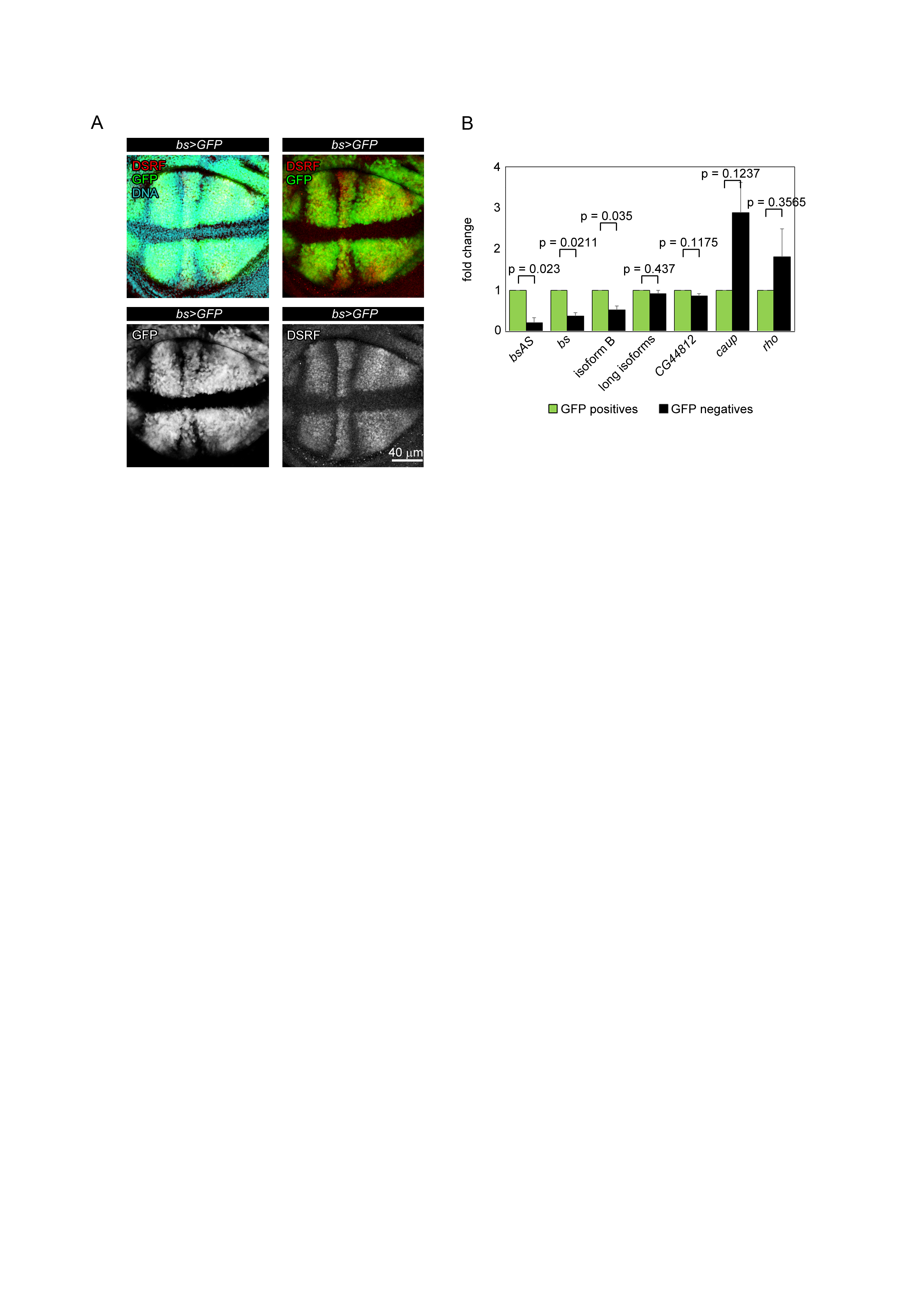

Supplement: S4 Fig — (A) Co-localization of GFP and DSRF in bs>GFP heterozygous third instar larvae imaginal discs. (B) Expression of bs isoforms and bsAS in intervein (GFP positive) and vein (GFP negative) regions. bsAS and the bs short isoform B are more expressed in interveins than in veins, which are marked by high expression of the vein-specific genes caupolican (caup) and rhomboid (rho). In contrast, the long isoforms of bs are expressed at comparable levels in veins and interveins. Statistical significance was computed by one-sample t-test. (TIF) [file pgen.1009245.s004.tif]

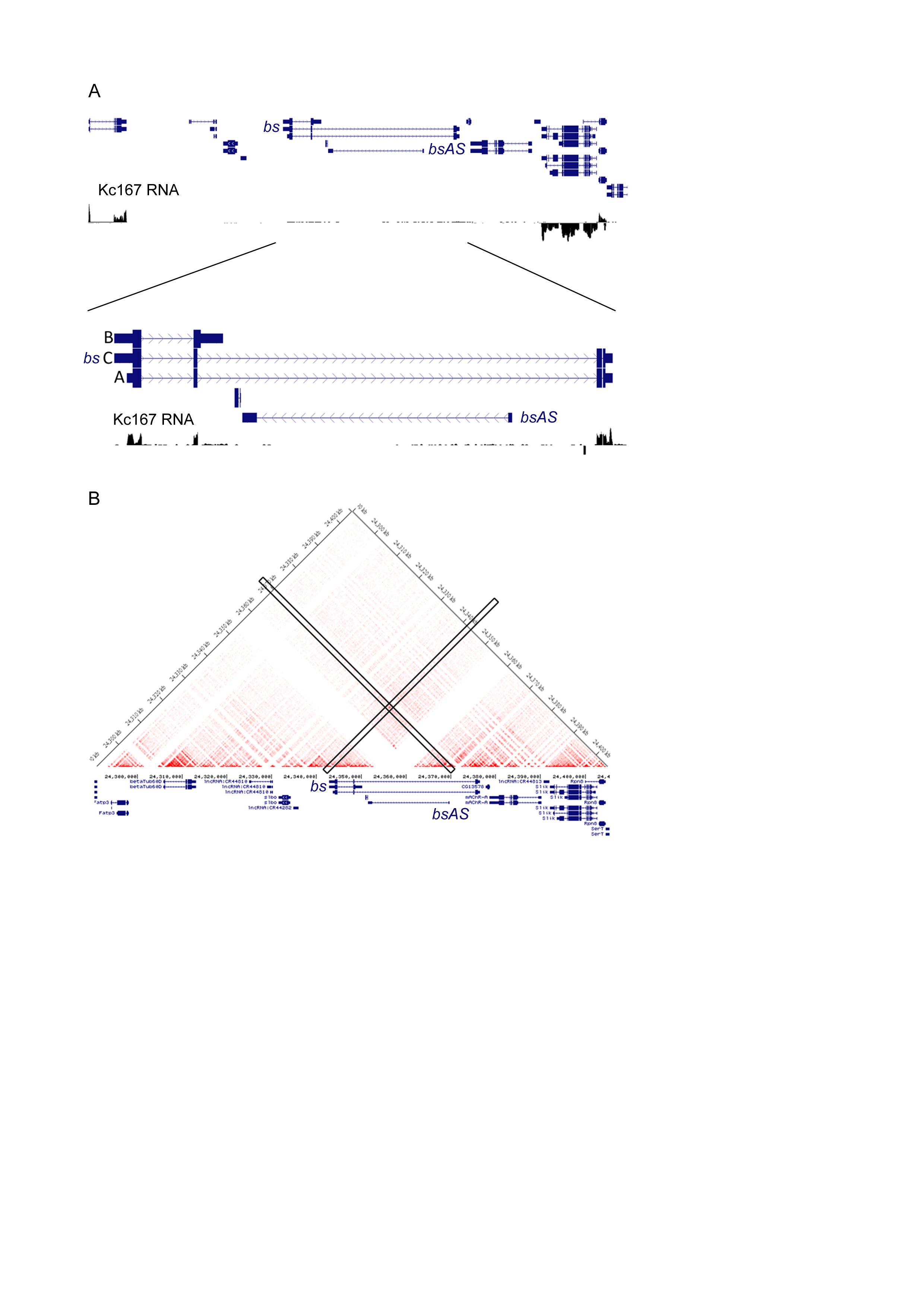

Supplement: S5 Fig — (A) RNA-Seq of Kc167 cells, from modENCODE [62]. bsAS is not expressed in these cells. bs expression is very low and isoform A seems to be the main expressed one. (B) Interaction of the bs locus and the neighboring regions (100 Kb). HiC data was obtained from Cubenas-Potts et al. [61]. The only strong interaction observed in this region is the contact between bsAS TSS and bs TSS1. No further interactions are observed. (TIF) [file pgen.1009245.s005.tif]

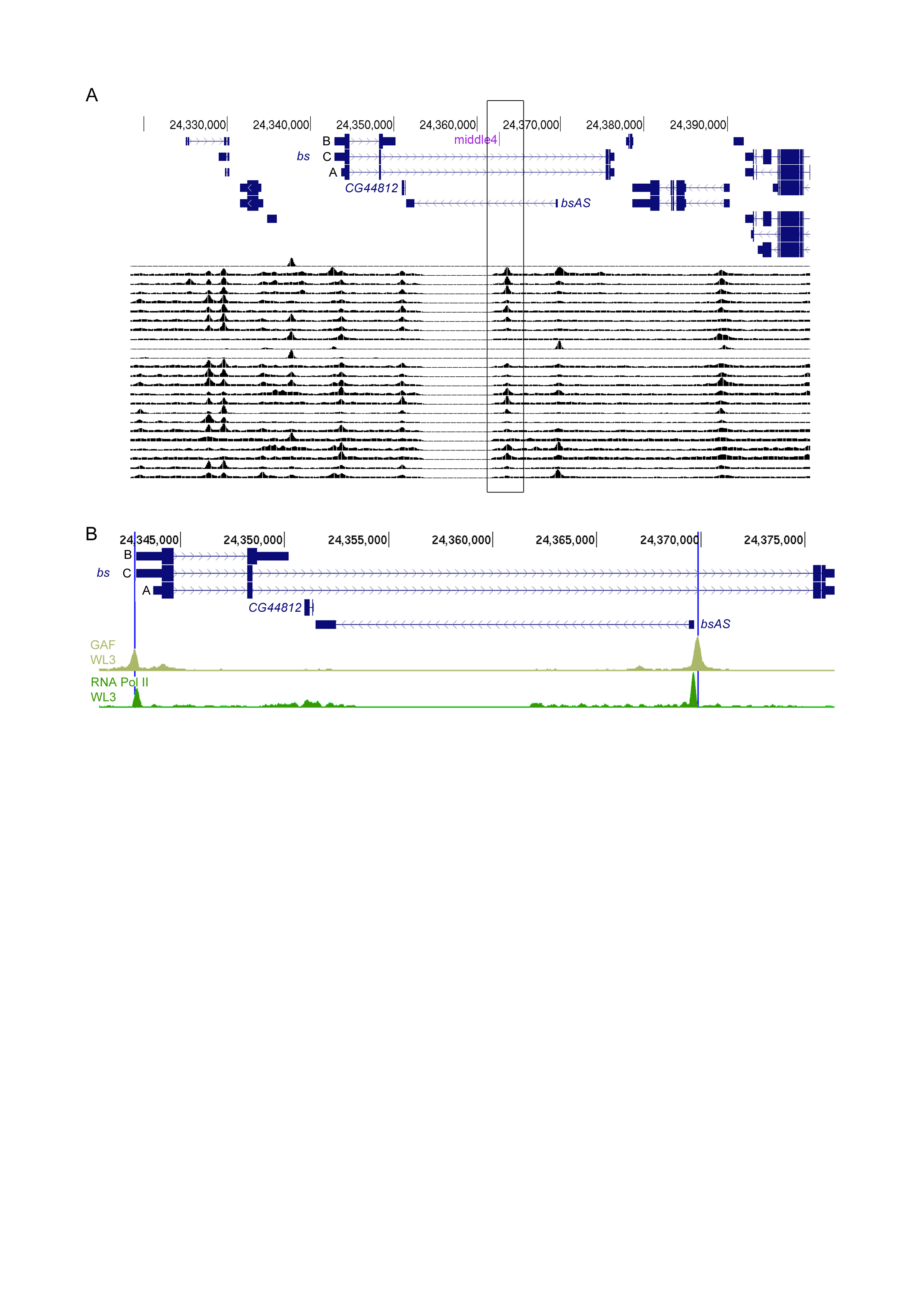

Supplement: S6 Fig — (A) Profile of DNA binding of 24 transcription factors during embryogenesis from modENCODE project [63]. Middle4 region is very close to a hot spot for transcription factor binding. (B) Profile of DNA binding of GAF [31] (light green) and RNA Pol II [32] (dark green) in third instar larvae wings. GAF and RNA Pol II peaks coincide with the expression of bs TSS1 and bsAS in wings. Blue vertical lines represent GAF binding sites at bs TSS1 and bsAS TSS. (TIF) [file pgen.1009245.s006.tif]

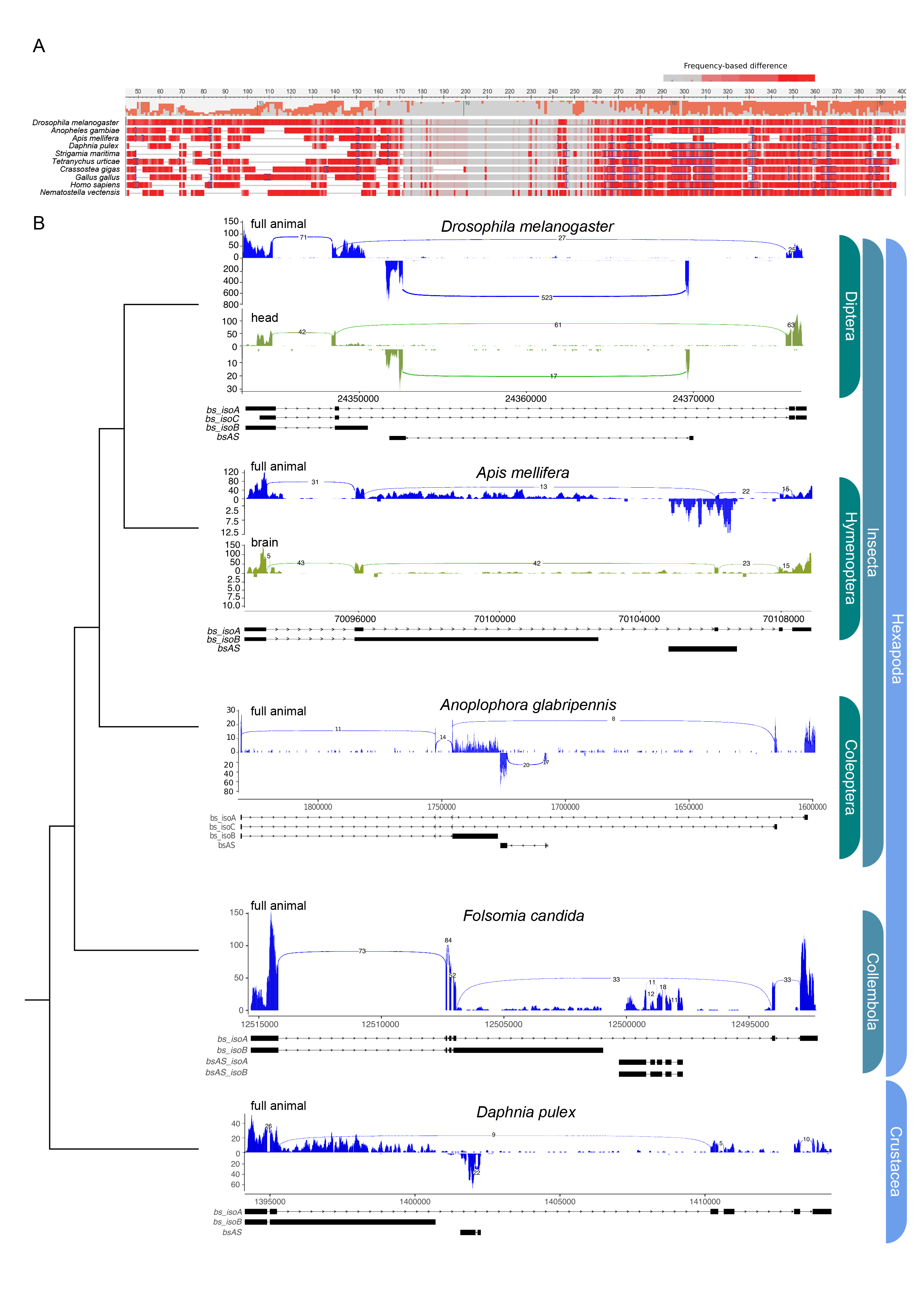

Supplement: S7 Fig — (A) Multiple sequence alignment of the long bs isoform using MAFFT and visualization of the frequency-based difference using NCBI MSA Viewer. High sequence conservation is observed between 160–270 bp where MADS-box is located. Sequence conservation drops rapidly outside this region. (B) Read-depth along bs locus. Organisms are sorted by the tree of life. Number of split reads are highlighted in the exon junctions generated using ggsashimi [39]. Stranded RNA-Seq is shown in two separated strands, where the negative strand is negated and shown below the positive strand. When available, whole animals (in blue) and heads (in green) RNA-Seq samples have been represented. The long isoform of bs is annotated in all species. Expression of both long and short isoforms of bs is supported by read coverage and by split alignments in exon junctions in represented species. Antisense expression is supported by stranded read coverage and by split alignments in the exon boundaries in the Diptera Drosophila melanogaster, in the Coleoptera Anoplophora glabripennis and in the Crustacea Daphnia pulex. Stranded RNA-Seq of the Hymenoptera Apis mellifera presents more antisense signal in the whole body compared to the head sample, however there are no split reads supporting exon junctions. The wingless basal Hexapoda, Folsomia candida, presents a clear set of split reads supporting the junctions of bsAS and no split reads shared between bs and bsAS, however the data is unstranded. Overall, Hexapoda and Crustacea show expression of both long-short bs isoforms as well as the antisense expression on the lncRNA bsAS. (TIF) [file pgen.1009245.s007.tif]
