# Supplementary material for: bsAS, an antisense long non-coding RNA, essential for correct wing development through regulation of blistered/DSRF isoform usage
Source: PLoS Genet. 2020 Dec 28;16(12):e1009245. doi: 10.1371/journal.pgen.1009245 (PMC7793246; doi:10.1371/journal.pgen.1009245)
Supplement: S1 Table — All samples generated along the manuscript are summarized here. (PDF) [file pgen.1009245.s008.pdf]

Sample	Total # reads	# Mapped reads	Proportion mapped reads	# Uniquely mapped reads	Proportion uniquely mapped reads
wtEAL3.1	92624048	91486808	98.7722	88894992	97.16701
wtEAL3.2	77533626	75828528	97.80083	73725748	97.22693
wtLL3.1	121021466	107715318	89.00513	105383724	97.83541
wtLL3.2	109413972	102201120	93.40774	97882412	95.7743
wtWL3.1	81030122	79816358	98.50208	78079790	97.8243
wtWL3.2	83521572	82426862	98.68931	78321148	95.01896
wtELP.1	134304082	129152790	96.16446	126591202	98.01662
wtELP.2	132043336	121297912	91.8622	118183372	97.43232
wtWLP.1	108100128	105568376	97.65796	102489310	97.08334
wtWLP.2	144086210	136518300	94.74765	133541172	97.81925
bsEAL3.1	147819126	136876976	92.59761	134435818	98.21653
bsEAL3.2	139808904	106272586	76.01275	85335706	80.29889
bsWL3.1	184430738	164711012	89.30779	160623490	97.51837
bsWL3.2	139062704	136397368	98.08336	133643534	97.98102
bsELP.1	210915056	201795336	95.67612	198332716	98.28409
bsELP.2	206900922	197297590	95.35849	193799048	98.22677
bsWLP.1	236383298	224236494	94.86139	220943980	98.53168
bsWLP.2	223774820	211371452	94.45721	207968748	98.39018
