# Supplementary material for: bsAS, an antisense long non-coding RNA, essential for correct wing development through regulation of blistered/DSRF isoform usage
Source: PLoS Genet. 2020 Dec 28;16(12):e1009245. doi: 10.1371/journal.pgen.1009245 (PMC7793246; doi:10.1371/journal.pgen.1009245)
Supplement: S2 Table — WL3, third instar larvae wing. WLP, late pupa wing. EAL3, third instar larvae eye-antenna. ELP, late pupa eye. Genes are highlighted in grey. Expression values are represented in TPMs. (PDF) [file pgen.1009245.s009.pdf]

		wt				*bsAS-/-*			
**gene**	**Gene/transcript**	**WL3**	**WLP**	**EAL3**	**ELP**	**WL3**	**WLP**	**EAL3**	**ELP**
blistered	FBgn0004101	25.545	48.175	7.81	3.87	72.68	120.79	9.885	9.405
	FBtr0072271 (A)	1.135	4.035	7.275	2.985	12.815	16.215	9.625	4.755
	FBtr0290089 (B)	18.89	42.21	0.535	0.645	16.13	34.965	0.06	0.845
	FBtr0343314 (C)	5.52	1.93	0	0.24	43.735	69.61	0.2	3.805
bsAS	FBgn0266046	60.185	108.79	2.145	1.585	0.03	0.075	0	0.02
	FBtr0343311	60.185	108.79	2.145	1.585	0.03	0.075	0	0.02
