# Supplementary material for: bsAS, an antisense long non-coding RNA, essential for correct wing development through regulation of blistered/DSRF isoform usage
Source: PLoS Genet. 2020 Dec 28;16(12):e1009245. doi: 10.1371/journal.pgen.1009245 (PMC7793246; doi:10.1371/journal.pgen.1009245)
Supplement: Attachment — Submitted filename: revision_answers.pdf [file pgen.1009245.s012.pdf]

Answers to the Reviewers:

Reviewer #1

In this work, Lluch and colabs searched for Natural Antisense Transcripts that could have a function during development by strand-specific RNA seq in different tissues of the fly. The authors identified bsAS as an antisense lncRNA to the bs/DSRF gene. bs is required for the proper vein-intervein pattern in the wing, trachea formation and also is implicated in neural processes such as memory, vision and sleep. Two different isoforms are described for bs. A long isoform (encoded by the A and C transcripts) and a short one (B transcript), being the later the one expressed in the wing disc. Interestingly, the authors beautifully demonstrated that bsAS is expressed in the same intervein pattern as bs in the wing and that bsAS is required in cis to determine which isoform is expressed. Mutants for bsAS showed a strong derepression of the long isoform without affecting the expression of the short one. Comparing the wing phenotypes of the bsAs mutants with the ectopic expression of the long isoform, the authors proposed that the intervein or neural cell fates are specified by the different usage of the bs isoforms. Moreover, using 3C assays demonstrated a physical contact between the transcription start site of bsAs and of the short isoform, suggesting a co-regulation. In addition, the authors perform an evolutionary analysis to propose a common origin of the bsAS and the long isoform structure of the bs gene.

The paper is well organized, written and the conclusions are novel and mostly well supported by their experiments. However, I have some discrepancies with some of the conclusions drawn from their results and few comments and questions that the authors may consider before publication.

We are glad that the reviewer found our work of interest, and the conclusions novel and mostly well supported by our experiments. We also want to apologize for the lack of clarity in parts of the text that may originate some of the issues raised by the reviewer. Thanks to the suggestions by this and the other reviewers, we believe that the revised version of our manuscript is more clearly written, and the conclusions more strongly supported.

1.1. One of my main problems with their model is that the authors conclude that the expression of the long isoform induces tissue neuralization (for example Fig. 5). In the text, the authors mistake the vein as a neural tissue misleading the reader in several paragraphs in the text (pg. 17 for example). Also, the long isoform is normally not expressed in the wing and therefore, unless it is proven, is not required for vein patterning. Is it reasonable to think, as suggested by the authors, that the different functions of bs are carried out by the different isoforms, however this hypothesis was not tested in the paper.

The reviewer is correct that veins cannot be fully considered neural tissue, but they contain the nerves that serve as sensory organs in insect wings (Fang et al., 2015). Thus, we expect some neural genes to be specifically expressed in the veins of the fly wing. We also agree that that the role of *bs* long isoform in promoting tissue neuralization cannot be fully concluded from our data. However, we do have strong indirect evidence that the long isoform of *bs* plays a role in neural development. First, the long isoform (A) is the only isoform expressed in the eye. Second, the mutation of *bsAS*, which induces the overexpression of *bs* long isoforms in the wing, promotes the overexpression of a number of neural-specific genes in this tissue. However, in the revised version of the manuscript, we have toned down the claim that the long isoform promotes tissue neuralization and we made very clear that we just hypothesize that it may lead to the activation of neural genes.

1.2. A simple way to interpret their results is that the expression of the long isoform in the bsAS mutants acts as a dominant negative version of the short isoform, generating a partial bs loss of function phenotype and the formation of extraveins.

Although we cannot rule out the possibility that *bs* long isoform acts as a dominant negative of the short one, we think that, actually, our results point in the opposite direction. We would like to note that the long isoforms are expressed at a similar level in the wing and in the eye, where no short isoform is present; this indicates that the long isoform can function independently of the short one. Actually, since expression of the short isoform could prevent tissue neuralization, the short isoform would be acting as a dominant negative version of the long isoform.

1.3 Maybe looking at vein patterning genes in the different mutant combinations could help understand the phenotypes and function of the different isoforms.

We have observed that very few changes occur at the third instar larvae stage, so all patterning experiments have to be performed in late pupal stages, where tissues cannot be fixed and permeabilized, so that antibodies cannot penetrate and immunostainings fail. We have tried to perform immunostainings in pupas 24-36 h after pupariation, as previously published (Classen et al., 2008), but the vein patterning did not show any phenotype, suggesting that the main changes in vein patterning occur, consistently with our RNA-Seq results, at later pupal stages.

1.4 Also, I think is important to show the expression of bs, of the two different isoforms and of bsAS in the different tissues such as wing vs eye –disc.

We agree with the reviewer, and we think that Table S2 provides this information. We have made reference to Table S2 more explicit in the ms, and have included a simplified version in Fig. 1D.

Gene/transcript	EAL3	WL3
blistered	7.81	25.545
isoform A	7.275	1.135
isoform B	0.535	18.89
isoform C	0	5.52
bsAS	2.145	60.185

Fig. 1D. Quantification of *bsAS* and *bs* isoforms in wt wings and eyes at third instar larvae (L3).

To further characterize the expression pattern of *bs*, and to complement the data in Table S2, in the revised version of the manuscript, we have performed additional experiments. In our original submission, we had already included the immunostaining of DSRF in the wing (Fig. 2C). Now, we also performed immunostaining in the eye. As seen in Fig. S3A, the level of the protein in the eye is too low to be detected in this way. Furthermore, we performed expression analyses of cells isolated from vein and intervein regions of third instar larvae wings *of bs·GAL4>UAS·GFP* flies (see Methods). The results indicate that, whereas *bsAS* and the short isoform of *bs* are mainly expressed in the intervein regions, *bs* long isoforms are

expressed at similar levels in veins and interveins (Fig. 3D). We have incorporated all these results in the manuscript.

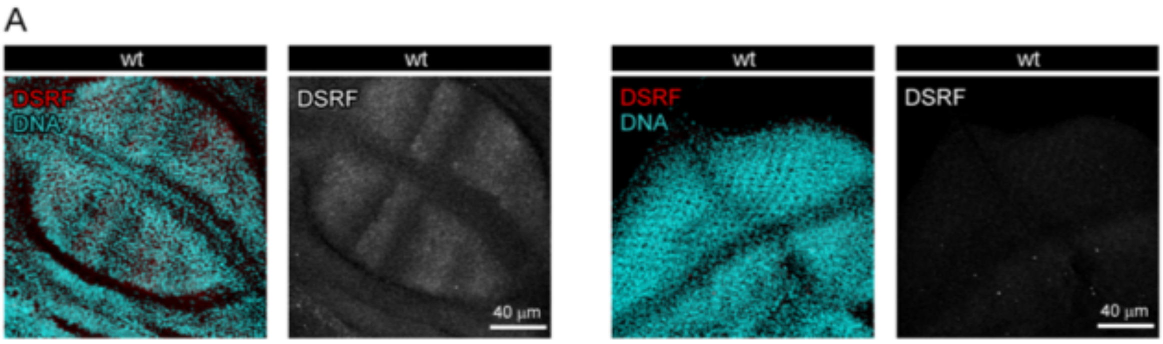

Fig. 2C and Fig. S3A. DSRF staining in wt wing (left) and eye (right) third instar larvae imaginal discs.

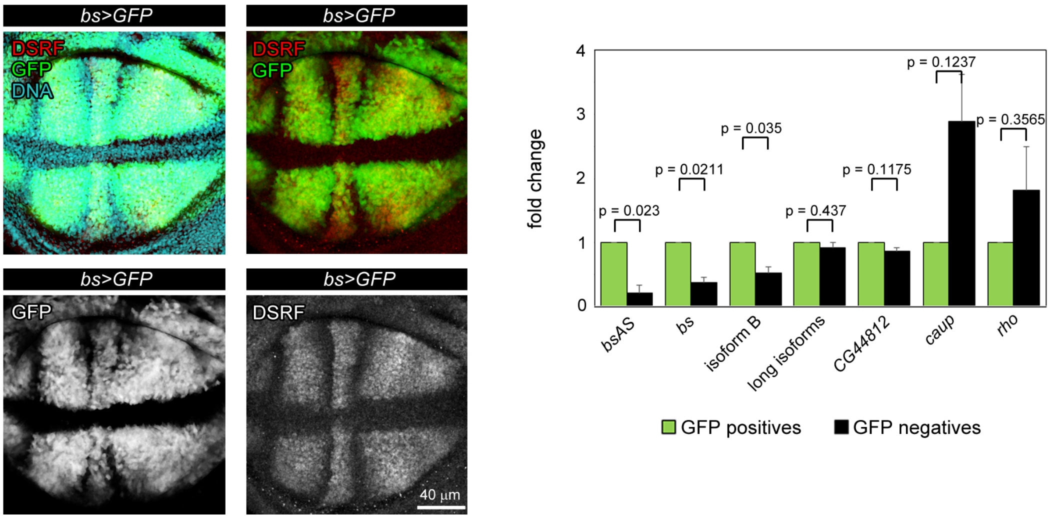

Fig. 3D. Expression of *bs* isoforms and *bsAS* in veins and intervein regions of *bs>GAL4* expressing wing imaginal discs.

2.1. In the discussion, the authors "hypothesize that bs short isoform can counteract the expression of the long isoforms in the intervein regions, silencing the expression of neural genes and avoiding the development of ectopic veins within intervein regions". However, if I understood correctly the paper and their results, is not the short isoform, but the transcription of the bsAS lncRNA the responsible for the downregulation of the long isoform in the intervein region. Again, the role of the long isoform inducing neural genes and ectopic vein development is not convincingly demonstrated.

The reviewer is correct. In the paper, we show that the transcription of *bsAS* lncRNA is responsible for the downregulation of the long isoform. We hypothesized that the short isoform could "counteract the expression of the long isoforms in the intervein regions", meaning that the short isoform of DSRF could act as dominant negative of the long one at protein level. We have clarified this in the manuscript.

2.2 It is a little confusing to me the different uses of isoform A or C in figure 5. They have shown that neither isoform are expressed in the wild type wing and only in the bsAS mutants, the isoform C is derepressed (pg. 14). So why picture iso A in the vein territories in fig. 5?

We apologize again by the lack of clarity. The long isoforms of *bs* are actually expressed in the wing (see Table S2). Our results suggest that *bsAS* and TSS1, which drives the expression of isoforms B and C, are co-expressed in intervein regions, while in the eye TSS2 is active, driving the expression of only isoform A. We are unable, however, to resolve the expression of *bs* isoforms in vein regions. Whether, as in the eye, TSS2 is the main promoter in veins, and therefore, only isoform A is produced. Or, in contrast, TSS1 is the main active promoter, but there is specific downregulation of *bsAS*, which cannot prevent the synthesis of the long isoform C. A third possibility is that both promoters are, at least partially, active. Given that we cannot distinguish which is the long isoform expressed in the vein, we have modified the model accordingly.

3. The upregulation of bs expression in the bsAS mutant is not evident at the RNA level in Fig. 2 and at the protein level it is hard to convince the readers comparing two different discs without any quantification. If the authors are convinced that there is an upregulation of Bs protein, maybe a WB could be more informative and quantitative.

Also, it is possible that the upregulation of the total bs levels is not detected because the derepression of the long isoform is very low compared to the short one, and therefore even an upregulation of the long one maybe masked and difficult to detect. I think this has to be clarified better.

In response to the reviewer concern, to further investigate *bs* upregulation, we have performed immunostainings in third instar larvae wings of wt, *bsAS* heterozygous and *bsAS* homozygous mutant wings. The experiments have been performed in parallel and images have been taken using the same settings in all experiments. The fluorescence detected in intervein region between veins 3 and 4 was quantified using imageJ. The results confirm that *bs* is overexpressed both in heterozygous and homozygous mutants (Fig. 2C-D). Actually, we also observed an increase in the signal in the vein regions, confirmed by the quantification of the fluorescence intensity of the vein 3. We have included these results in the revised version of our manuscript.

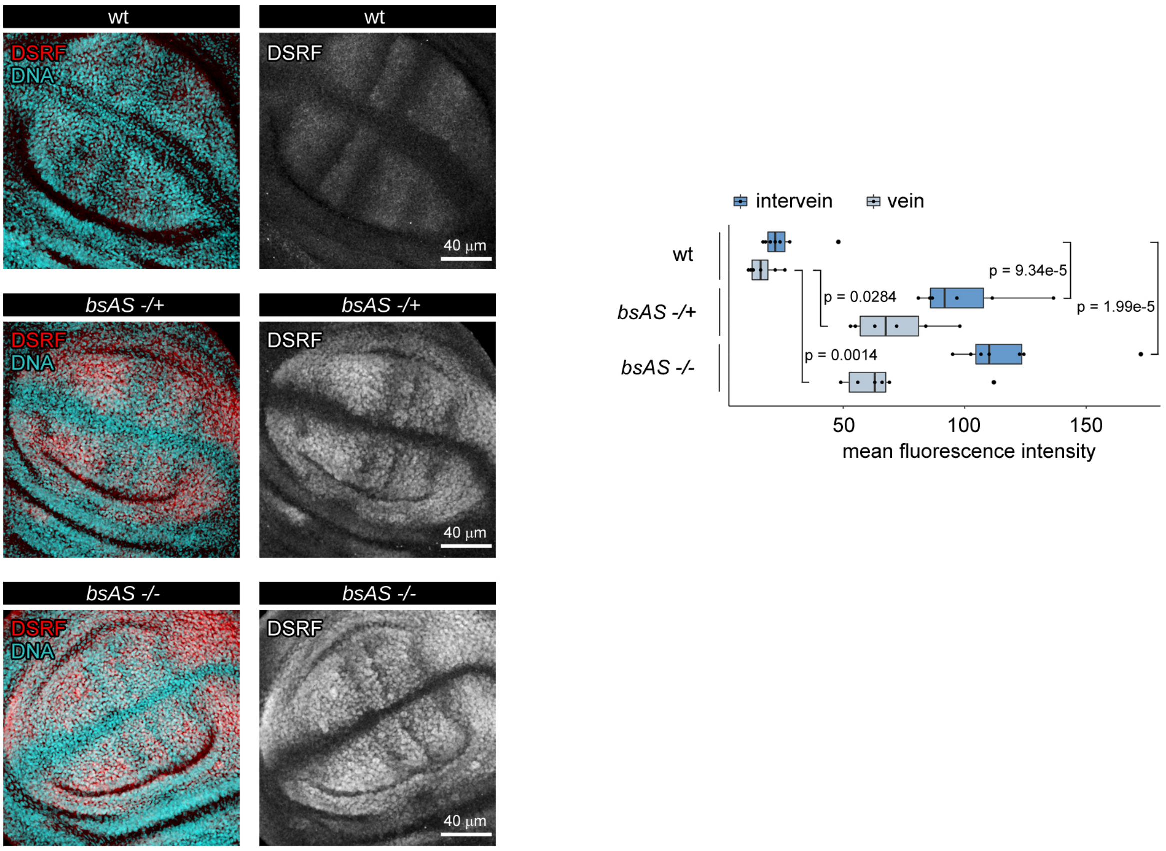

Fig. 2C-D. DSRF staining and quantification in wt and *bsAS* mutant wings at third instar larvae.

4. In a bs mutant the entire intervein domain is transformed into vein tissue (see Fig. 2C Roch et al, 1998). This phenotype is different from what is observed in the homozygous mutant for bsAS where some extra vein tissue is observed but most of the intervein region is preserved. I don't think the phenotypes are equivalent because in the bsAS mutant the short isoform is still normally expressed. The authors conclude that the bsAS mutant phenotype is a consequence of the overexpression of the long isoform. To demonstrate this important conclusion the authors could use specific RNAis available to the long isoforms such as JF02319 and KK108659 in the bsAS mutant background to test if the bsAS mutant phenotype could be restored.

We thank the reviewer for this suggestion. We have performed the recommended RNAi rescue experiment We have performed this rescue with JF02319 (B26755) flies, as this transgene is inserted into chromosome III and, thus, compatible with the *bsAS* mutation. The results indicate that the overexpression of the RNAi is able to rescue the wild type phenotype on a heterozygous *bsAS* background, and partially rescue the homozygous phenotype (Fig. 2J and Fig. S3G). We have included these results in the manuscript.

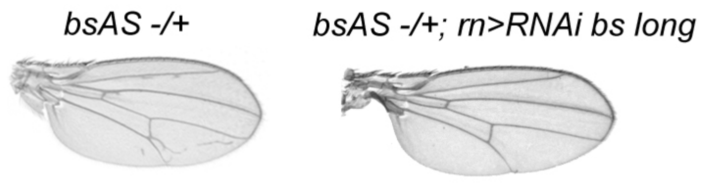

Fig. 2F,J. Rescue of wild type phenotype on *bsAS -/+* wings by overexpressing an RNAi against the long isoform of *bs*.

5. Do the authors find any vein promoting gene upregulated in their RNA seq of bsAS mutant wings? How is the pattern of genes such Knirps (kni) and Iroquois (Iro) in bsAS mutants?

The RNA-Seq data show that these genes are indeed overexpressed in the *bsAS* mutant, however, they do not not pass the thresholds of fold-change and FDR established.

	WL3	
	**wt**	***bsAS -/-***
knirps	11.96	13.96
araucan	42.48	42.64
caupolican	25.96	32.92

Table R1. Expression of vein patterning genes in third instar larvae wing imaginal discs, in TPMs.

6. The authors showed a co-regulation of bsAS and the TSS1 using 3C techniques. As both bs and bsAS are expressed in the same intervein pattern, are they regulated by the same cis-regulatory module? Is there a physical interaction between those regions with the identified wing CRM of bs (Nussbaumer et al, 2000)?

According to the high-resolution HiC maps from Cubenas-Potts et al., apart from the interaction between *bsAS* TSS and *bs* TSS1, no more interactions occur between *bsAS* or *bs* TSSs and any region 100 kb around, even in the region corresponding to the CRM described in Nussbaumer *et al*, located between *bs* and *slbo* genes. This figure has been included in the supplementary material (Fig. S4B).

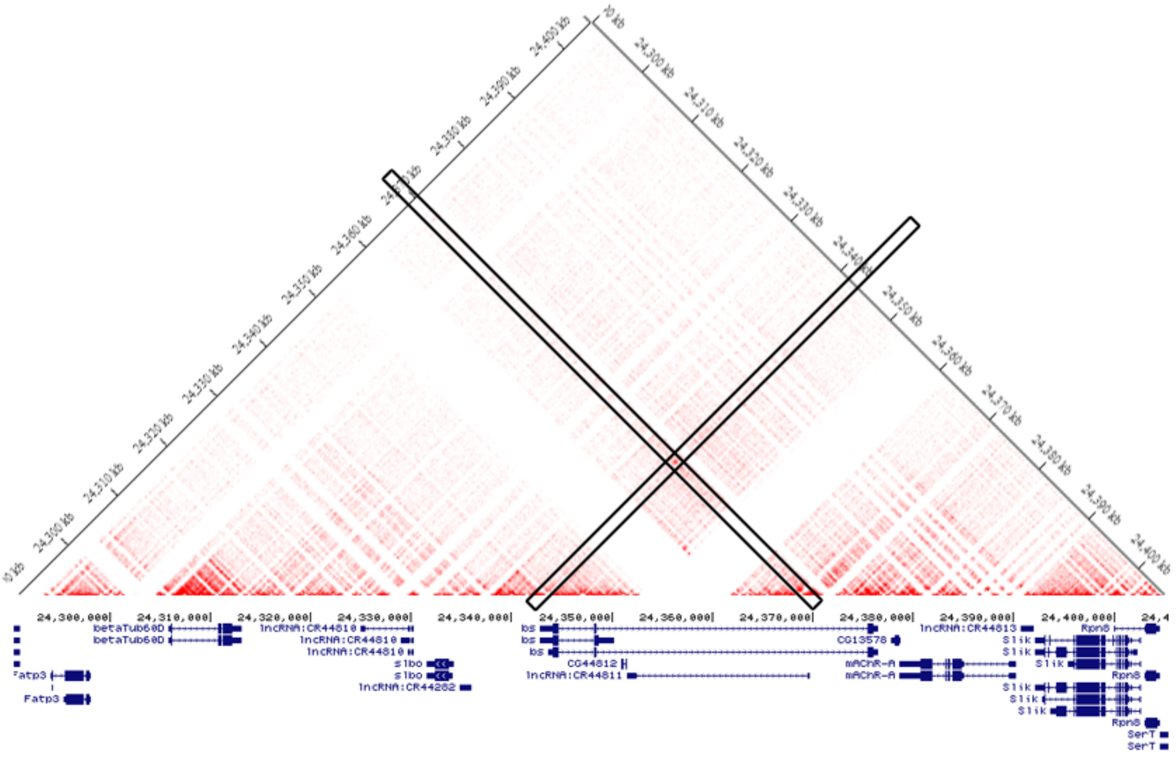

Fig. S4B. Interaction map of *bs* locus (Cubenas-Potts et al., 2017).

Minor comments:

-p Value in Figure 1D and 2B for CG4812.

We have included it.

-is bs expressed in the EA disc? Maybe including a picture could be informative.

We know that the long isoform of *bs* is expressed in this tissue according to our RNA-Seq data (Table S2). In the revised version of the manuscript, we are now including the immunostaining of DSRF in the eye (Fig. S3A, see response 4.1). Furthermore, we would like to note that we have also performed immunostaining in the *bsAS* mutants. Even though no phenotype is observed in adult stages, the *bsAS* homozygous mutants show a localized overexpression of DSRF in some determined cells posterior to the morphogenetic furrow (Fig. S3A). These results have been included in the manuscript.

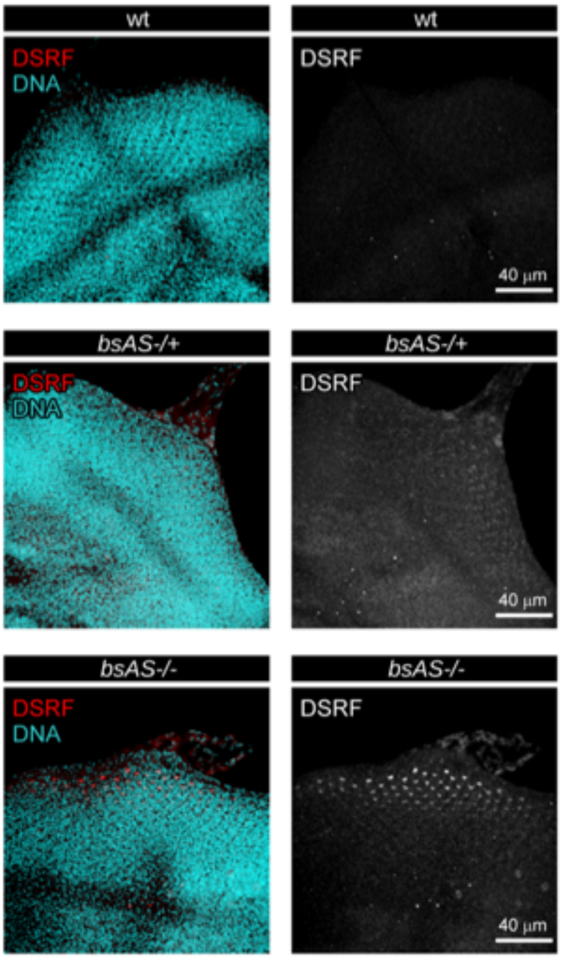

Fig. S3A. DSRF staining in wt and *bsAS* mutant eye imaginal discs.

-The comparation and p values are not clear in Fig. 3C and D.

We have modified this figure and we hope they are now clearer (Fig. 4C-D).

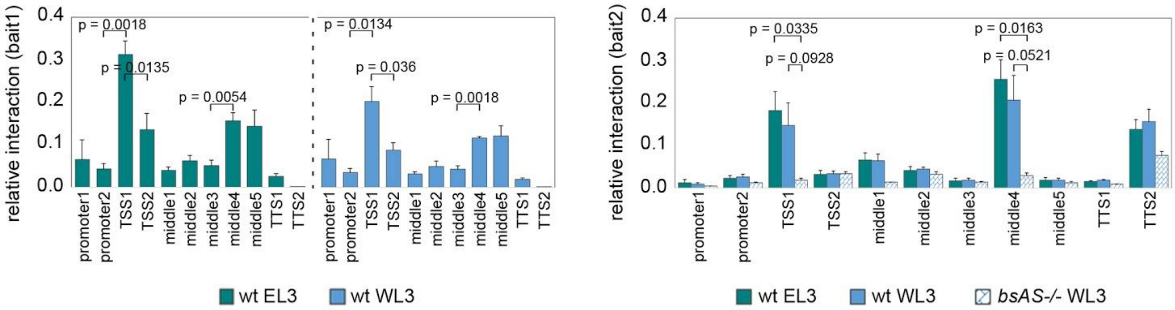

Fig. 4C-D. Interactions of *bsAS* and *bs* TSSs in wing and eye tissues.

-Wl3 track for bs expression in Fig. Sup2I. The expression of the bs isoforms is not observed. Compare it with Fig. 1C. Why?

This was due to the different track scale. Given that, in *bsAS* mutants, *bs* is strongly overexpressed, we set a different scale than in Fig. 1 to compare the isoform usage in these mutants vs wt. We have now fixed it to make it clearer (Fig. 3A).

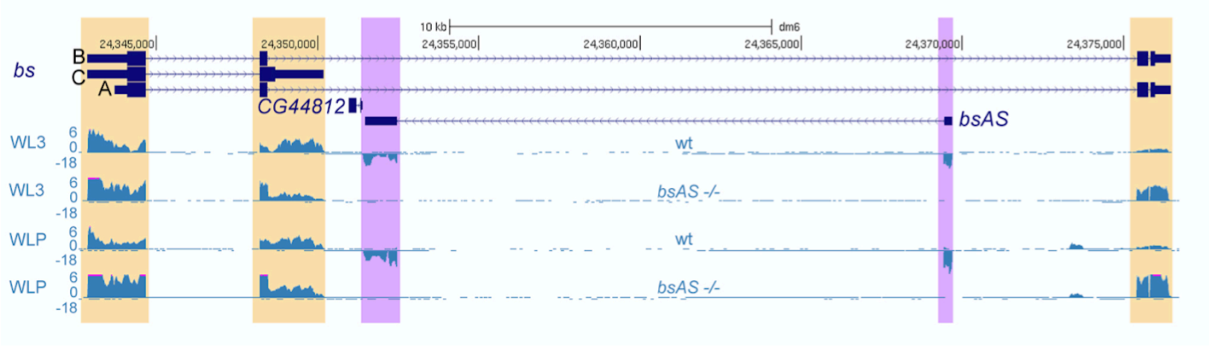

Fig. 3A. Expression of *bs* locus in wt and *bsAS* mutants in third instar larvae and late pupa stages.

Reviewer #2

In this study, Perez-Lluch et al., investigate the role of an antisense long non-coding RNA (lncRNA) in regulating usage of the bs/DSRF transcripts and the subsequent role in wing development. First, they describe the characterization of lncRNAs. First they identify the bs/DSRF gene as a potential candidate for transcript usage depending on lncRNA. Comparison of bs/DSRF transcript usage reveal strong differences in wing versus leg or eye-antennal discs. bs/DSRF has been shown to be expressed in the presumptive intervein tissue of the wing disc and the antisense transcript (bsAS) appears to be expressed in a similar pattern. Using a CRISPR/CAS9 strategy they make a specific deletion of the bsAS. This deletion results in the strong increase in the expression of the bs/DSRF long transcripts and in severe wing phenotype. The authors interpret this phenotype as a consequence of high bs/DSRF long transcript; however when over-expressing the bs/DSRF long transcript they observe minor (Fig 2H) or no visible phenotype (Fig S2E-F). Next, they show direct interactions between the transcription start site of bs and bsAS. Finally, the author analyze throughout evolution the conservation of the dual transcripts of the bs/DSRF orthologues. Experiments are properly performed to address the issue of the bsAS function. However there a major problem with the interpretation. The bsAS deficiency induces an extreme phenotype that is not at all comparable to the one induced by over-expression of the bs/DSRF long transcript. Thus, they cannot postulate that the extreme wing phenotype is due to the increased expression of the bs/DSRF long isoform. The conclusion (p14 bottom) and the model (Fig5) are over-estimated. I doubt that this study can be published in PLoS Genetics, unless they provide a consistent explanation based on experimental data for the severe wing phenotype observed in bsAS deficiency.

We are glad that the reviewer found that our experiments are properly performed. We agree with the referee that the phenotype observed in wings overexpressing the long isoform of *bs* was, indeed, milder than the phenotype of *bsAS* mutation. However, further analyses point to the fact that the phenotype of *bs* long isoform may be dose dependent, as we found that slightly increasing the temperature at which flies grow (from 23-24 ºC to 25 ºC), induces a stronger phenotype in the wing. Also, the usage of *nubbin* driver promotes a stronger phenotype in the

adult (Fig. 2I and Fig. S3F). In the revised version of the manuscript, we included the results of the overexpression of the *bs* long isoform at 25ºC and using the *nubbin* driver (Fig. 2I and Fig. S3F).

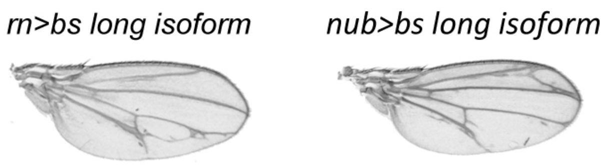

Fig. 2I and Fig. S3F. Adult male wings of flies overexpressing the long isoform of *bs* under the control of different drivers.

While stronger than reported in our original submission, the phenotype observed with the overexpression of *bs* long isoform is still milder than that observed in *bsAS* homozygous mutants. We believe that this is partially due to our inability to overexpress this isoform at levels comparable to those observed in the *bsAS* mutant. We have quantified the expression of the long isoform of *bs* and found the levels of the protein to be significantly lower than those of the *bsAS* mutant wings, even when compared to heterozygous mutants (Fig. S3D). Even so, the phenotype observed for *m>bs long isoform* wings is actually stronger than that observed for heterozygous mutants (compare Fig. 2I with Fig. 2F).

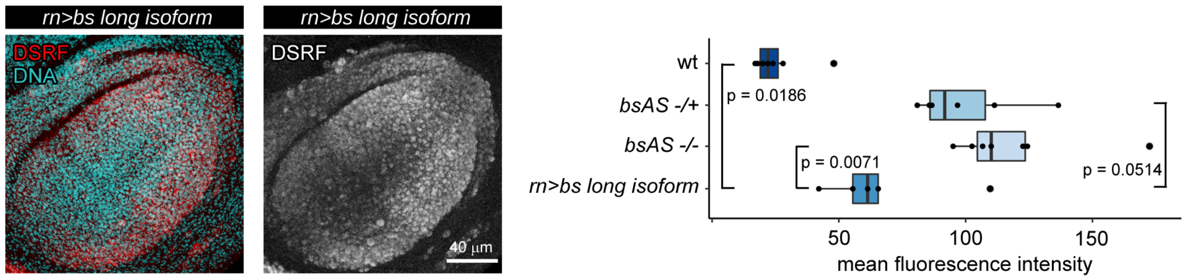

Fig. S3D. Expression of DSRF in wt, *bsAS* mutant and *bs* long isoform overexpressing third instar wing imaginal discs.

Additional comments:

1. Ref13 (Montagne et al., 1996) should be mentioned as the same time as ref10 (Fristrom et al., 1994) since ref13 is the one showing that bs and DSRF are allelic.

We have included it.

2. References for fly strains in M&M must be provided.

We have included it.

3. In situ hybridization in wing disc for bsAS and bs are not the “same’, they exhibit an overlapping pattern. The text should be modified.

We have fixed it in the text.

4. The author cannot compare Fig2C (immunostaining to bs/DSRF) with Fig1E (in situ hybridization). Actually, there is no control for the genuine protein level in wild type disc in Fig2C.

The control for DSRF expression was actually represented in Fig. 1E. The upper panels of this figure represented DSRF immunostaining in wt discs. To make it clearer, we have included the wt and *bsAS* DSRF immunostainings in the same figure (Fig. 2C).

5. bs/DSRF protein levels should be compared in the same experiment for wild type, for bsAS deficient and for long transcript-expressing wing discs.

We have performed this comparison, and we have seen that there is a significant overexpression of *bs* both in heterozygous and homozygous *bsAS* mutant wings, whereas the overexpression of *bs* long isoform under the *rotund* driver is lower (Fig. S3D). We have included these results in the revised version of the manuscript.

6. The phenotype of bsAS heterozygous deficiency is minor, nonetheless, resembles the one of bs/DSRF long transcript over-expression; the author should also compare bs/DSRF protein levels in both genetic context.

The phenotype of the *bsAS* heterozygous wing is very variable. Still, the comparison of the DSRF protein levels between these wings and wings overexpressing the *bs* long isoform shows that DSRF is higher expressed in *bsAS* heterozygous wings than in *rn*>*bs* long isoform ones (Fig. 2C-D). We have included these results in the revised version of the manuscript.
