# Supplementary material for: bsAS, an antisense long non-coding RNA, essential for correct wing development through regulation of blistered/DSRF isoform usage
Source: PLoS Genet. 2020 Dec 28;16(12):e1009245. doi: 10.1371/journal.pgen.1009245 (PMC7793246; doi:10.1371/journal.pgen.1009245)
Supplement: Attachment — Submitted filename: Answers_to_the_reviewers.pdf [file pgen.1009245.s013.pdf]

Answers to the reviewers

Reviewer #1:

I acknowledge the effort and the experiments done by the authors to respond to the reviewers comments and suggestions. Overall, I really like the paper and it deserves to be published in PloS Genetics. The experiments proven the role of the LncRNA bsAS as a regulator of bs isoform usage are very solid.

We are very glad that the reviewer finds our work of interest. We acknowledge his/her comments that have helped to improve our manuscript.

However, I still identify some problems with the interpretation of the data that were raised in the first revision.

1. Specifically, I find the role of the bs long isoform as a determinant of vein fate during normal development not been proved. Moreover, the authors insists in consider the vein tissue as a neural tissue and in my opinion this is an inaccuracy and could lead to confussion.

The reviewer is correct that the veins themselves are not neural tissue, but the nerves that run along them are. In the reviewed version of the manuscript, we thought that we have already acknowledged this. In this revised version, we have further modified the introduction to clarify that veins are “epithelial formations that carry the trachea and nerves of the adult wing”. Besides, we have also reviewed the discussion section to clarify the role of *bs* long isoform in the wing. In this sense, we agree that the role of *bs* long isoform in the determination of neural fate has not been proven; still, the fact that this isoform of *bs* is the only one that is expressed in the eye tissue, altogether with the knowledge that *bs/DSRF* has been related to memory and learning in *Drosophila* points into that direction.

2. To prove the role of the bs long isoform as a determinant of vein tissue in the wing, the authors should demonstrate:

2.1) bs long isform expression in the vein region. In situ hybridization of the bs long isoform?

We agree that this experiment would help understanding the expression pattern of *bs* long isoform in the wing. However, the level of expression of the long isoform at the third instar larvae wing is very low (6.6 TPMs), making the detection by *in situ* hybridization not feasible.

Actually, the experiment added in the revised version of the manuscript (Fig. 3D) pretends to answer this question (see response 4).

2.2) the funtion of the bs long isform in a wt background in the wing. A simple experiment would be to knockdown the bs long in the wing with an specific RNAi. If their model (fig. 6) is correct, the prediction would be wings with defective veins. However, in fig. 2J (although no ideal as it is performed in the bs+/- background) no defect on the characteristic vein pattern is observed.

We have performed the experiment proposed by the reviewer but no phenotype was observed in the vein patterning, likely due to the robustness of the system. This observation could argue in favor of the role of the long isoform of DSRF as dominant negative of the short one; however, we think that the fact that the long isoform is expressed in regions where the short one is absent, such as the eye disc, and that *bs* gene has been associated to neural behaviors, such

as memory and learning, suggests that the long isoform of *bs* may be involved in neural development. Nevertheless, we have modified the text and the model accordingly.

3. Sentences like “In contrast, in the vein regions, in which bsAS is poorly expressed, the long isoforms (A or C, or both) are the dominant ones, inducing the expression of neural genes and the differentiation of veins” and their model in fig. 6 lead to a function of the long isform as determinant of vein fate that has not been proven.

We have reviewed the discussion clarifying the putative role of *bs* long isoforms in the wing and have modified the model accordingly.

4. The authors also include a new experiment where cells GFP+ and GFP- cells are isolated of third instar larvae wings of bs-GAL4>UAS-GFP flies and conclude that the bsAS and short isoforms are expressed mainly in intervein (GFP+) while long isoforms are expressed at similar levels in vein and intervein. GFP- cells are formed by vein cells (minority) and hinge and notum cells (mayority), and therefore this experiment is not very precise, informative and conclusive.

We agree with the reviewer’s observation that the GFP negative cells do not correspond only to veins, but also to hinge and notum cells. However, we think that the experiment is actually very informative, given that the higher expression of a vein specific gene, such as *rho*, in GFP negative cells indicates that the sample is, indeed, enriched in cells belonging to vein regions. However, given that the experiment is not fully conclusive, we have moved the figure to the supplementary information.

Reviewer #2:

The revised version of the manuscript of Perez-Lluch et al., is more convincing than the first one. My main concerns have been addressed; in particular, immunostaining pictures that strengthen the relationship between bsAS activity and bs protein level expression, and novel data showing that selective knockdown of the bs/DSFR long isoform can significantly suppress the wing phenotype of bsAS-/- mutants.

We are glad that the reviewer finds the revised version of the manuscript much improved over our original submission.

Minor point: In the discussion, the authors stress the ancestral conservation of bs and bsAS throughout evolution, even in wingless animals, and conclude by an original function in neural differentiation. However, wings have been proposed to have evolved from ancestral gills (doi.org/10.1038/385627a0). Further, dSRF plays a critical role in the differentiation of the terminal tracheal cells (Guillemin et al 1996 Development 122:1353-62). Therefore, one can also speculates that the ancestral conservation of bs and bsAS may rely on an original function in the development of the respiratory system. Although it would be easy to look at the differentiation of the terminal tracheal cells in bsAS mutant flies, I consider that it is not the purpose of the present study. Nonetheless, I suggest a modulation of the neural differentiation origin in the conclusion.

We thank the reviewer for making us aware of this possible evolutionary scenario, which we had not previously considered. Following the reviewer’s recommendation, we have modified the discussion, toning down the potential neural origin of the differentiation, and including this alternative explanation.
